# Neuroprotective Effect of Antioxidants in the Brain

**DOI:** 10.3390/ijms21197152

**Published:** 2020-09-28

**Authors:** Kyung Hee Lee, Myeounghoon Cha, Bae Hwan Lee

**Affiliations:** 1 Department of Dental Hygiene, Division of Health Science, Dongseo University, Busan 47011, Korea; kyhee@dongseo.ac.kr; 2 Department of Physiology, Yonsei University College of Medicine, Seoul 03722, Korea; mhcha@yuhs.ac; 3 Brain Korea 21 PLUS Project for Medical Science, Yonsei University College of Medicine, Seoul 03722, Korea

**Keywords:** neuroprotection, antioxidant, brain, neurodegenerative disease, oxidative stress

## Abstract

The brain is vulnerable to excessive oxidative insults because of its abundant lipid content, high energy requirements, and weak antioxidant capacity. Reactive oxygen species (ROS) increase susceptibility to neuronal damage and functional deficits, via oxidative changes in the brain in neurodegenerative diseases. Overabundance and abnormal levels of ROS and/or overload of metals are regulated by cellular defense mechanisms, intracellular signaling, and physiological functions of antioxidants in the brain. Single and/or complex antioxidant compounds targeting oxidative stress, redox metals, and neuronal cell death have been evaluated in multiple preclinical and clinical trials as a complementary therapeutic strategy for combating oxidative stress associated with neurodegenerative diseases. Herein, we present a general analysis and overview of various antioxidants and suggest potential courses of antioxidant treatments for the neuroprotection of the brain from oxidative injury. This review focuses on enzymatic and non-enzymatic antioxidant mechanisms in the brain and examines the relative advantages and methodological concerns when assessing antioxidant compounds for the treatment of neurodegenerative disorders.

## 1. Introduction

Oxidative stress is considered as a detrimental condition for normal brain functioning. Since the brain uses chemically diverse reactive species for signal transmission, it is susceptible to oxidative stress [1]. The human brain consists of more than 86 billion neurons and over 250–300 billion glial +cells, which consume over 20% of the total basal oxygen [2,3]. The mitochondria in the brain use the inspired basal oxygen to reduce O_2_ to H_2_O to support adenosine triphosphate (ATP) synthesis [4]. The electron transport chain in the mitochondria is not efficient and leakage of reactive oxygen species (ROS) from the mitochondria occurs when there is excess of oxygen [5]. Redox signaling in the brain functions as an intrinsic sensor to oxidative stress, when signals go awry during pathological conditions. Brain tissue has a high rate of oxidative metabolic activity, intense production of reactive oxygen metabolites, relatively low levels of antioxidants, low repair capacity, non-replicating neuronal cells, and a high membrane surface to cytoplasm ratio [6]. The cellular antioxidant system that prevents tissue damage is composed of endogenous and exogenous antioxidants that have the ability to reduce different chemicals. In biological antioxidant defense systems, both endogenous and exogenous antioxidants are classified into enzymatic antioxidants and non-enzymatic antioxidants, including oxidative enzyme inhibitors, antioxidant enzyme cofactors, ROS/RNS scavengers, and transition metal chelators [7,8]. These two main antioxidant systems play an important role in maintaining the balance between pro-oxidant and antioxidant agents in the brain and in mitigating oxidative stress [9]. The present review focuses on enzymatic and non-enzymatic antioxidant mechanisms in the brain and examines the relative advantages of antioxidant compounds for the treatment of various brain diseases. Therefore, this review will describe selective antioxidants shown to have neuroprotective effects that limit neurodegenerative disease.

## 2. Factors That Contribute to Vulnerability of the Brain to Oxidative Stress

**Figure 1:**
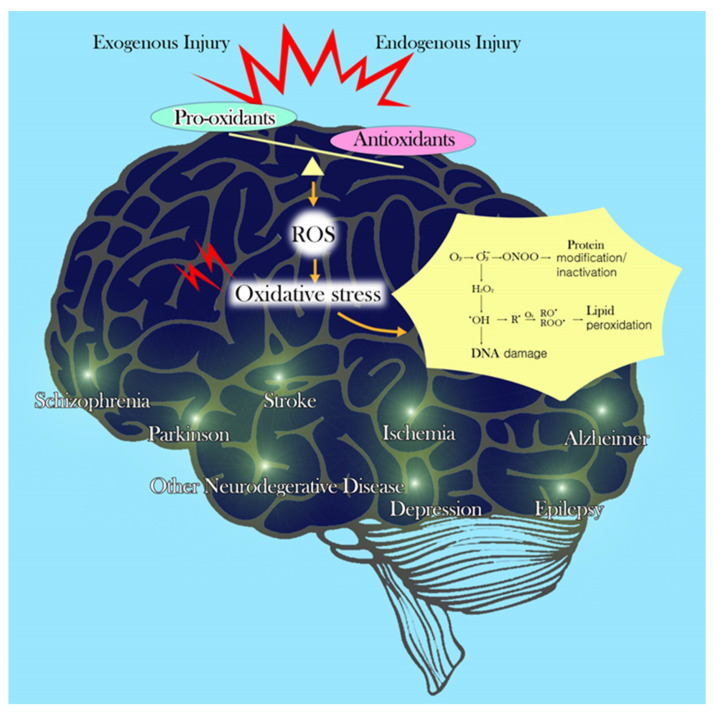
Development of various diseases by the pathophysiology of oxidative stress in the brain. Balance and imbalance between pro-oxidants and antioxidants against reactive oxygen species production induce oxidative stress and are consequently involved in neuronal damage resulting in the neurodegenerative diseases.

Free radicals or other reactive molecules disturb cellular energy metabolism and cause oxidative stress. Endogenous redox imbalance by pro-oxidant and antioxidant agents occurs as a result of free radicals, which play an important role in oxidative stress, cell death, and tissue damage. Increased free radical production due to excess pro-oxidant mechanisms can react with lipids, proteins, nucleic acids, and other biomolecules which can alter their structure and function. A high concentration of polyunsaturated fatty acids exists in membrane lipids in the brain [10]. These are sources of decomposition reaction in the form of lipid peroxidation, in which a single initiating free radical can precipitate the destruction of adjacent molecules. Polyunsaturated fatty acids especially serve as major biological targets for oxidative damage induced by ROS. Other candidate molecules that serve as biological targets of free radicals are nucleic acids. Breaks in DNA or modified bases can result in aberrant gene expression and cell death [11]. Moreover, free radicals can also oxidize the backbone and side chains of proteins, thereby disrupting the function of enzymes, receptors, neurotransmitters, and structural proteins by oxidative modification [12]. Highly reactive oxygen radicals are produced in the brain as a result of interactions between various transition metals and their reducing equivalents. Transition metals including iron, copper, zinc, and manganese, are associated with increased free radical production by the Fenton reaction [13,14]. Since the brain has abundant lipid content, high energy requirements, and weak antioxidant capacity, it is an easy target for excessive oxidative insults. Specifically, ROS increase susceptibility to neuronal damage and functional decline via brain oxidation in Alzheimer’s disease (AD), Parkinson’s disease (PD), amyotrophic lateral sclerosis (ALS), cerebrovascular disorders, psychiatric disorders, and other neurodegenerative diseases [6,13,15] (Figure 1).

Considering a detailed perspective on signaling, the rationale underlying the vulnerability of the neurons in the brain to oxidative stress are as follows: In redox signaling, O_2_^−^/H_2_O_2_ generation by the reduced form of nicotinamide adenine dinucleotide phosphate (NADPH) oxidase 2 induces the activation of signaling proteins via sulfenic acid formation [16,17]. The brain is dependent on Ca^2+^ signaling for synaptic plasticity, a fundamental brain function, and it expends considerable amounts of ATP to maintain intracellular Ca^2+^ homeostasis. Synaptic terminal glutamate-induced Ca^2+^ transients activate neuronal nitric oxide synthase (nNOS)-mediated NO^·^ generation, and additionally, Ca^2+^ overloaded in the mitochondria induces O_2_^−^/H_2_O_2_ generation which can lead to ONOO^·^ production and excitotoxicity [18,19,20]. Particularly, the mitochondria generate O_2_^−^ in complex I and complex III and the monoamine oxidase enzyme isoform catalyzes H_2_O_2_ generation during metabolism [21,22,23]. The human brain consumes over 25% circulating glucose to support neuronal function; however, protein inactivation by the formation of advanced end glycation products (AGE) due to decreased glycolytic rates can cause glucose-induced oxidative stress [24,25]. Neurotransmitters with a catechol group render the brain sensitive to oxidative stress. Redox-active transition metals catalyze the auto-oxidation of dopamine to a semiquinone radical during neurotransmitter oxidation [26,27,28]. The mature neurons of the brain have abundant and multiple mechanisms that promote long-term neuronal survival and prevent cell death; however, the brain has a comparatively weak endogenous antioxidant defense system relative to other tissues and is susceptible to imbalance in redox homeostasis. For instance, neurons with 50 times lower catalase content than hepatocytes have constrained glutathione peroxidase 4 (GPX4) activity (owing to low glutathione [GSH] content) and a modest antioxidant defense mechanism [29,30,31]. Microglia monitor neuronal activity for removal of unhealthy cells, neuronal wiring during development, and activity dependent synaptic plasticity. They generate O_2_^−^ via NADPH oxidase 2 (NOX2) within an end-foot process, thus influencing brain oxidation [32,33]. Redox-active transition metals, such as Fe^2+^ and Cu^+^, are enriched in the brain and therefore contribute to ferroptosis by catalyzing peroxyl (ROO^·^) and alkoxyl (RO^·^) radical generation [34,35]. Lipid peroxidation, which involves initiation, oxygenation, propagation, and termination, occurs within the neuronal cell membrane [36]. The brain uses nNOS and NOX isoforms to maintain essential functions. However, nNOS/NOX expression is associated with NO^·^ generation, which can be spatially co-generated while producing ONOO^·^ [37,38]. In summary, these interconnected, myriad factors render the brain vulnerable to oxidative stress. Furthermore, excessive production of reactive species and insufficient activity of antioxidant defense systems have been implicated in the pathogenesis of neurodegeneration.

## 3. Role of Antioxidants in the Brain

Antioxidants act in two ways: First, the antioxidant defense systems, which are initiated against oxidative damage, prevent generation of ROS, and block and capture the generated radicals [39]. These systems are present in the aqueous and membrane compartments of cells; they can be enzymatic or non-enzymatic. The first line of defense has been identified as an enzymatic antioxidant system that includes superoxide dismutase, catalase, and glutathione peroxidase. The second line of defense is represented by reduced thiol and non-enzymatic antioxidants, including hydro- and lipo-soluble or metabolic compounds [7]. Second, during the antioxidant repair process, the removal of damaged biomolecules before their aggregation causes alterations in cell metabolism. The intervention by the repair systems consists of repairing oxidatively damaged nucleic acids by specific enzymes, removing oxidized proteins by the proteolytic systems, and repairing oxidized lipids by phospholipases, peroxidases, or acryl transferases [8,39,40].

Antioxidants act to directly scavenge oxidizing radicals and regenerate oxidized biomolecules in organisms. Neuronal cells possess several factors that minimize oxidative damage and a complex antioxidant system consisting of various antioxidant enzymes and non-enzymes. With regard to the mechanism of action of antioxidants, antioxidants respond to different steps of oxidative radical processing and can be considered by taking into account the different steps of lipid peroxidation in cell membranes such as: initiation, propagation, and chain termination [36]. The first step of initiation of lipid peroxidation can be promoted by diverse exogenous factors, endogenous enzyme systems, and the electron transport chain in the mitochondria. The propagation step of peroxidation is initiated by the addition of oxygen to carbon-centered radicals, which are generated at, or near, the diffusion-controlled rate. Propagation is represented by the transfer of a hydrogen atom to the chain carrying peroxyl radicals and occurs at a relatively slow rate. Antioxidants can inhibit lipid peroxidation by removing molecular oxygen or by decreasing the local concentration of molecular oxygen, removing pro-oxidative metal ions, trapping aggressive ROS, scavenging chain initiating radicals, breaking the chain of free radical reactions, and quenching singlet oxygen [7,41]. In its protective role, enzymatic antioxidants are chain-breaking antioxidants which are able to scavenge radical species while non-enzymatic antioxidants are singlet oxygen quenchers, metal chelators, oxidative enzyme inhibitors, peroxide decomposers, and/or ultra violet (UV) radiation absorbers [7]. Additionally, antioxidant enzymes can catalyze the synthesis or regeneration of non-enzymatic antioxidants.

### 3.1. Enzymatic Antioxidants

#### 3.1.1. Superoxide Dismutase

**Figure 2:**
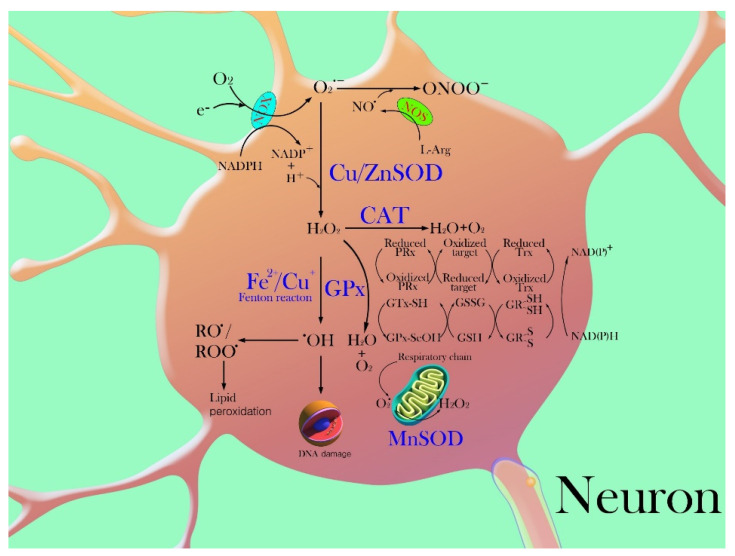
Enzymatic antioxidant defense system against the production of reactive oxygen species (ROS). Various pathways of cell death caused by ROS and its transformation are observed in brain injury. NADPH oxidase and mitochondrial respiratory transport chain are known as major cellular sources of the superoxide radical anion (O_2_^−^). The superoxide radical anion reacts with nitric oxide (NO) to form the peroxynitrite anion (ONOO^−^) which mediates oxidative modification of protein residues via an interaction with NO. The superoxide radical is dismuted by the superoxide dismutase enzyme (SOD) to form hydrogen peroxide. In addition, manganese containing superoxide dismutase (Mn-SOD) reduces the superoxide radical anion generated during the electron transport chain in the mitochondrial matrix. Catalase (CAT) and/or glutathione peroxidase (GPx) decomposes hydrogen peroxide to water and oxygen by enzymatic reactions. Hydrogen peroxide is decomposed into reactive hydroxyl radicals by reaction with catalytically active redox metals such as (copper and iron). Hydroxyl radicals can react with oxygen to form peroxyl or alkoxyl radicals which can lead to lipid peroxidation and react with DNA and primarily cause damage to DNA. Peroxiredoxins (Prx) and thioredoxin (Trx) act as redox-regulated proteins to additional redox relay bases.

Superoxide dismutase (SOD), which belongs to the enzymatic defense system, changes the superoxide radical anion to H_2_O_2_ by oxidative decay. Another function of SOD is to protect dehydratase from free radical superoxide inactivation. There are three forms of SOD in humans. SOD-1, a copper-zinc containing SOD (Cu, Zn-SOD), is present in the cytosol and specifically catalyzes dismutation in a pH-independent medium [42]. SOD-1 protein is a homodimer composed of eight antiparallel beta strands and two metal atoms that catalyze the conversion of toxic O_2_^−^ anions to H_2_O_2_ and O_2_. Specifically, copper minerals play a crucial role in the catalytic function of this enzyme, while zinc is important for structural integrity [43]. SOD-1 plays an important role as a first line of defense by detoxifying superoxide radicals. In addition, the lack of this enzyme exhibited a pronounced susceptibility to paraquat toxicity. SOD-2, a manganese containing SOD (Mn-SOD), is found in the mitochondrial matrix and reduces the superoxide radical anion generated in the electron transport chain [44]. A detailed analysis revealed that the SOD-2 subunit needs one metal atom and that it is functionally disabled in the absence of Mn atoms in the active site. SOD-2 attracts O_2_^−^ and turns positive on the active side. The active metal provides electrons directly to O_2_^−^ then reduces one molecule of O_2_^−^ and one proton to be converted to form H_2_O_2_ (Figure 2). The amount of SOD-2 is different in each cell type, depending on the number of mitochondria. Specifically, the presence of SOD-2 is essential for the survival of aerobic cells and the development of cellular resistance to oxidative stress. SOD-3, an extracellular SOD (EC-SOD), also contains copper and zinc in its structure, is synthesized inside the cell and secreted into the extracellular matrix [45]. SOD-3 enzyme is not induced by its substrate or other oxidants and is regulated to coordinate with cytokines, rather than as a response of individual cells to oxidants [46].

In neurodegenerative diseases, SOD-1 and SOD-2 genes appear to be disrupted and/or mutated. SOD-1 gene mutations produce diverse cellular changes such as alteration of gene expression, unusual protein interactions, caspase activation, mitochondrial dysfunction, and cytoskeletal abnormalities in familial ALS [47]. Overexpression of SOD-1 protects neurons against the neurotoxic effects of amyloid beta (Aβ), and loss of SOD-1 accelerates aging-related pathologies and reduces lifespan in mice [48,49,50]. SOD-1 deficiency in an AD model showed that it was associated with augmented Aβ oligomerization and memory impairment mediated by oxidative stress [51]. Overexpression of SOD-2 by treatment with sodium orthovanadate could rescue synaptic failure and neuronal cell death in the hippocampus after kainic acid (KA)-induced oxidative stress; moreover, an increase in SOD-2 protected the neuronal death from oxidative injury [52]. Impaired SOD-2 is a common potential pathogenesis related to oxidative stress in PD and AD [6]. In human amyloid precursor protein (hAPP) transgenic mice with over 50% reduced SOD-2 activity, increased SOD-2 protects the age-related brain against hAPP/Aβ-induced impairments [53]. Extracellularly administered SOD was effective in inhibiting cell death and restoring healthy mitochondrial morphology in a monosodium glutamate-induced excitotoxicity disease model [54]. An increase in SOD-2 can be expected to assist or improve neuronal function and vascular pathology in AD as a therapeutic effect.

#### 3.1.2. Catalase

Catalase (CAT), a heme-containing tetrameric protein is a common antioxidant enzyme naturally produced by the body when exposed to oxygen. H_2_O_2_ generated inside the cell is enzymatically catabolized in aerobic organisms by catalase and the activity of several peroxidases. CAT is one of the most efficient enzymes that cannot be saturated by H_2_O_2_ at any concentration. It reacts with H_2_O_2_ to form water/alcohol and oxygen using Fe as a cofactor [55,56]. CAT protects cells by detoxification of the generated H_2_O_2_ and plays an important role in the acquisition of tolerance to oxidative stress as an adaptive response [57]. CAT can maintain the concentration of O_2_ either for repeated rounds of chemical reduction or for direct interaction with the toxin [58] (Figure 2). Furthermore, inhibition of CAT activity results in enhanced cytotoxicity and increased ROS, indicating an important role of CAT in maintaining the oxidative balance [59]. Specifically, mislocalized CAT is associated with accumulation of H_2_O_2_ [60] and other ROS in the cells leading to compromised neurological function [61]. Thus, deficiency or malfunction of CAT is postulated to be related to the pathogenesis of many age-associated degenerative diseases.

CAT treatment reduces H_2_O_2_ levels and improves neuronal survival following Aβ-induced toxicity in neuronal culture [62]. In Aβ toxicity, CAT–SKL (serine-lysine-leucine) treatment reduced the pathology of microglial activation, and rat brains did not show long-term memory impairments via the reduction of H_2_O_2_ levels [63]. In the PD model, mutant α-synuclein inhibited the expression and activity of CAT and induced low catalase activity and high H_2_O_2_ production [64]. This antioxidant enzyme plays an important role in maintaining oxidative balance.

#### 3.1.3. Glutathione Peroxidase

Glutathione peroxidase (GPx) catalyzes the reduction of a variety of hydroperoxides (ROOH and H_2_O_2_) to water or the corresponding alcohols using GSH to protect mammalian cells against oxidative damage [65] (Figure 2). GPx has four identical subunits containing one selenocysteine in each residue for essential enzyme activity, which are classified as selenium-containing GPxs (GPx1–4 and 6) and their non-selenium congeners (GPx5, 7, and 8) [30]. The glutathione redox cycle is mostly active during low levels of oxidant stress, while CAT is more significant in protecting against severe oxidant stress. GPx isoenzymes appear to have antioxidant functions at different locations and cellular compartments, and the levels of expression of each isoform vary depending on the type of tissue, even though their expression is omnipresent. Furthermore, GPx is responsible for the conversion of GSH to oxidized glutathione disulfide (GSSG) and glutathione reductase (GR) reduces GSSG back to GSH [66]. GPx1 and GPx4 are found in most tissues and the predominant forms of GPx are found in brain tissue. GPx1 reduces H_2_O_2_ and organic hydroperoxides and is expressed both in neurons and astrocytes [67]. GPx4, the phospholipid hydroperoxide glutathione peroxidase, is located in the cytosol and a membrane fraction that can directly reduce phospholipid hydroperoxides, fatty acid hydroperoxides, and cholesterol hydroperoxides [68]. In the brain, mitochondrial and cytosolic GPx4 isoforms have been detected in neurons of the cerebral cortex, hippocampus, and cerebellum. In contrast, GPx4 in glial cells is barely activated under normal physiological conditions, whereas reactive astrocytes upregulate the expression of GPx4 following selective brain injury [31]. Recently, GPx4 has become well known as a key regulatory factor in ferroptosis which is a non-apoptotic and iron-dependent programmed cell death pathway that causes a rapid elevation of oxidative stress [69]. As the function of GPx is related to normal development and cellular metabolism via the regulation of oxidative stress, GPx4 function is potentially the key for cell survival.

GPx activity decreased significantly in PC12 cells during oxygen-glucose deprivation [70], and a two-fold increase in GPx and a three-fold increase in CAT activities were observed during a time course that reflected the temporal increase in nerve growth factor (NGF) in a brain contusion model [71]. The ferroptotic potential of neurons in the forebrain regions of the hippocampus and cerebral cortex is severely affected in AD patients, with increased vulnerability to ferroptosis. The role of GPx4 as a key regulator of ferroptosis was observed in Gpx4BIKO transgenic mice, a mouse model with a conditional deletion of GPx4 in forebrain neurons. Gpx4BIKO transgenic mice showed significant deficits in spatial learning and memory function and exhibited hippocampal neurodegeneration compared to control mice [72]. In human PD tissue biopsies, GPx4 expression was upregulated and ferroptosis-related events were observed [73].

#### 3.1.4. Thioredoxin

The thioredoxin (Trx) system consists of two types of antioxidant oxidoreductase proteins, Trx and thioredoxin reductase (TrxRs) with NADPH as an electron donor. Trx has a conserved active site such as Cys-Pro-Gly-Cys, that has an important function of acting as an active oxidoreductase and electron donor of some peroxiredoxins (Prx) which are crucial for the reduction of peroxides [74]. Trx is an important regulator of cellular function that can respond to redox balance by modulating signaling pathways, transcription factors, and immunological responses for cell survival in many conditions, including neurodegenerative diseases [75]. There are three isoforms in the Trx family such as Trx1 in the cytosol, Trx2 in the mitochondria, and a testis-specific Trx3 in mammalian cells. Trx1 exists in cell compartments such as the nucleus and the plasma membrane or as a secreted protein, depending on its localization and function in different cell types [76]. Trx1 is coupled with Prx 1/2 and methionine sulfoxide reductase and is essential role in the control of growth and apoptosis. TrxR 1 and Trx 1 are observed in neuronal synaptic vesicles. In addition, Trx 1 and 2 expressions are also present mainly in rat brain neurons. Neurons with mitochondrial dysfunction by complex IV inhibition show low levels of Trx and are thus, more vulnerable to H_2_O_2_. Reduced Trx is a powerful reductase that acts through a disulfide–dithiol exchange mechanism to catalyze the conversion of disulfide bonds into thiols with high efficiency [77]. The disulfides in the oxidized Trx are converted to thiols by the consumption of NADPH [78] (Figure 2). TrxR, a homodimer, catalyzes the reduction of the disulfide at the Trx active site and is encoded by three distinct genes: the cytosolic TrxR (*TrxR1*), mitochondrial TrxR (*TrxR2*), and thioredoxin-glutaredoxin reductase (*TrxR3*). TrxR can directly reduce substrates such as peroxides, including lipid hydroperoxides, H_2_O_2_, and protein disulfide isomerase [76,79]. TrxR is also involved in the regeneration of other antioxidant molecules such as dehydroascorbate, lipoic acid, and ubiquinone [80,81,82]. TrxR is very important when other selenoproteins including GPx lose most of their activity and it effectively donates electrons during DNA synthesis [83]. TrxR activation in the rat brain can be maintained at a certain level under severe selenium (Se)-deficient conditions. Both Trx and TrxR are widely expressed in tissues and organs, including the brain. In a central nervous system (CNS) study, it has been suggested that increased expression of Trx and TrxR is closely associated with cell damage due to oxidative stress [84,85]. In addition, NADPH and human TrxR are efficient electron donors to human plasma peroxidase and reduce hyperoxides, even with low GSH capacity [86].

The upregulation of Trx was observed as a neuroprotective effect on retinal ganglion cells against oxidative stress-induced neurodegeneration [87]. Overexpression of Trx1 attenuated endoplasmic reticulum stress by regulating the activation of the molecular mechanism for neuroprotection in an in vitro and in vivo model of PD [88]. In AD brains, TrxR activity was increased in the cerebellum and amygdala [89]. It has been implied that TrxR activation increases a compensatory mechanism during increased oxidative stress that is limited by the substrate Trx and the subsequent neurodegeneration seen in AD. Another evidence for the peripheral response to oxidative stress during neurodegeneration is the reduction of Trx1 and TrxR1 in the plasma and erythrocytes in blood samples from patients with Huntington’s disease (HD) [90]. Exogenously administered human recombinant Trx attenuates the generation of ROS involved in cytotoxic mechanisms, ameliorates neuronal damage, and augments neurogenesis following brain ischemia/reperfusion injury in rats [91]. In the ischemic brain, administration of Prx3 and Trx2 shows substantial neuroprotective effects by reducing oxidative stress [92].

Important enzymatic antioxidants such as SOD, CAT, GPx, and Trx remove superoxide and peroxides before they react with metal catalysts to form more reactive species (Figure 2). Peroxidative chain reactions initiated by reactive species that escape enzymatic degradation are terminated via chain-breaking antioxidants, including water-soluble ascorbate, lipid-soluble vitamin E, and ubiquinone. To enhance the antioxidative effect, oxidative stress should be controlled by supplying all the antioxidant nutrients and by minimizing the effect of substances that stimulate reactive oxygen metabolites (ROM) [93].

### 3.2. Non-Enzymatic Antioxidants

#### 3.2.1. Antioxidant Enzyme Cofactors (Selenium, Coenzyme Q10, Zinc)

##### Selenium

Selenium (Se) is an enzymatic cofactor and an essential component of selenoaminoacids and selenoproteins. Se has numerous biological functions related to redox signaling, antioxidant defense systems, thyroid hormone metabolism, and humoral and cell-mediated immune responses [94]. Se reacts with redox signals involving one- or two-electron transitions; however, it is less reactive and prefers higher oxidation states. The biological activity of Se varies depending on its concentration: normal growth and development at low concentrations, homeostatic function at moderate concentrations, and induced toxicity at high concentrations [95]. The complex biological functions of Se are found in general body proteins that mainly and effectively act through 25 selenoproteins that have selenocysteine at their active center in humans [96]. Over half of the selenoproteins exhibit antioxidant activity, of which the GPx family and Trx reductase family are well known. Selenoproteins constrain the activation of nuclear factor-κB (NF-κB) via redox signaling, which prohibits a cytokine storm and the formation of reactive oxygen and nitrogen species. Se is essential for brain function, but the Se expression level in the brain is rather poor compared to other tissues [97,98]. The distribution of Se in the human brain enriched in gray matter tended to be higher, while the white matter was found to have reduced Se levels. Specifically, Se level was observed to be highest in the hippocampus, cerebellum, and brainstem in the rat brain [99]. The mechanism of Se neuroprotection is attributed to modulation of Ca^2+^ influx via ion channels, anti-inflammatory effect by abrogation of microglia invasion, and biosynthesis stimulation of antioxidative selenoproteins in the brain [100,101].

Se is essential for the brain and plays an important role in the pathology of neuronal disorders such as AD, PD, ALS, and epilepsy [96]. In Se-deficient transgenic mice, Aβ plaques showed more than a two-fold increase compared to Se-adequate ones [102]. Selenite administration in a rat AD model also showed attenuation of cognitive deficits, oxidative damage and morphological changes in the hippocampus and cerebral cortex [103]. In a paraquat induced PD model, bradykinesia and DNA damage reduction were observed by supplementing Se with selenite in drinking water [104]. Alterations of Se homeostasis in the HD model have been observed and a beneficial effect of selenite supplementation in mice expressing mutant huntingtin has been reported. Additionally, autopsy of the human HD and mouse HD brain showed decreased Se content [105]. Selenite administration prevents secondary pathological events, thus, contributing to the reduction of apoptotic cell death and prevention of neuronal destruction in the cortex and hippocampus of traumatic brain injury [106,107]

##### Coenzyme Q

Coenzyme Q (CoQ) is a remarkable liposoluble ubiquinone with a long isoprenoid side chain and a main component of the internal mitochondria membrane, Golgi complex membrane, and lysosomal membrane. CoQ is an endogenous free radical scavenger and enzyme cofactor produced by most human cells and is abundant in the brain and intestine in mitochondria, the site of oxidative phosphorylation [108]. It is biosynthesized by all membranes and a component of the mitochondrial respiratory chain, which participates in electron transport [109]. CoQ in either reduced form, hydroquinone or ubiquinol, is a potent lipophilic antioxidant and is also involved in the regeneration and recycling of other antioxidants such as tocopherol and ascorbate [110]. CoQ is an important antioxidant that protects cellular membranes and lipoproteins against the toxic effects of free radicals that are generated during general metabolism [111]. The redox functions of CoQ mainly occur in the mitochondria as electrons shuttle between complexes I and II of the respiratory chain. Dehydrogenases oxidize NADH, NADPH, and dihydroflavine-adenine dinucleotide (FADH2) and transfer protons and electrons to ubiquinone, which is converted to ubiquinol. Finally, the protons transfer to the mitochondrial matrix and the electrons move to the cytochromes. Thus, cytochromes reduce the superoxide radical anion to water with electrons and protons from the matrix. These processes are required to produce ATP [108]. TrxRs within the cytosol induce the reduction of CoQ to ubiquinol and maintain the extra-mitochondrial antioxidant defense [112]. CoQ is synthesized by at least 12 proteins that form a multiprotein complex in the mitochondria, and the pathogenesis of CoQ10 deficiency involves deficient ATP production and excessive ROS formation in humans. However, CoQ10 deficiency is unique among mitochondrial disorders because an effective treatment is available by oral CoQ10 supplementation, to which many patients respond well [113,114]. However, dietary supplementation is still challenging due to the low bioavailability of the compound. Because CoQ10 levels decline with age, it has been studied in a variety of neurodegenerative disorders and aging but with disparate results [115].

In KA-induced oxidative injury in vitro [116], an increased number of surviving CA3 neurons was observed at 0.1 and 1 µM concentrations in CoQ10-treated groups using cresyl violet staining. CoQ10 (0.01, 0.1, and 1 µM) treatment significantly decreased 2,7-dichlorofluorescein fluorescence, and the expression of NQO1 in the CoQ10-treated groups was significantly lower than that in controls, indicating the protective role of CoQ10 in hippocampal neurons against oxidative stress. Rats exposed to intrastriatal CoQ10 showed a larger number of dopaminergic neurons, higher expression of neurogenetic and angiogenetic factors, and less inflammation, and these effects were more prominent than the orally administered CoQ10. Thus, continuous intrastriatal administration of low doses of CoQ10 showed a more effective strategy to prevent dopaminergic neuronal degeneration in a PD rat model induced by 6-hydroxydopamine [117]. In patients with acute ischemic stroke, a significant increase in CoQ10 level was observed with the administration of CoQ10 (300 mg/day group) compared with the placebo group. CoQ10 supplementation attenuated oxidative stress and neuroinflammatory marker levels and improved the neurological outcome scale and antioxidant enzyme activity [118]. In PD patients, the total Unified Parkinson’s Disease Rating Scale (UPDRS) scores decreased in the CoQ10-treated group, indicating amelioration of the symptoms and significant improvement in wearing-off in PD [119]. CoQ10 increased a reduced glutathione and SOD levels in patients with pregabalin-treated fibromyalgia. The supplementation of CoQ10 effectively reduced pain sensation, anxiety and brain activity, mitochondrial oxidative stress, and inflammation [120].

##### Zinc and Essential Metals

Zinc, a redox inactive metal, does not directly interact with ROS but has a crucial role in maintaining redox balance for the antioxidant defense system in various ways in the cell. Zinc increases the activation of antioxidant enzymes such as SOD, GPx, and CAT. It also acts as a direct cofactor of SOD-1 and SOD-3 and as an indirect cofactor for GPx [121]. Zinc inhibits important pro-oxidant enzymes such as NADPH oxidase, inducible nitric oxide synthetase (iNOS), and the reduced form of nicotinamide adenine dinucleotide (NMDA) and regulates oxidant production and metal-induced oxidative damage. Zinc is dynamically associated with sulfur in protein cysteine clusters. It mediates the induction of the zinc-binding protein metallothionein which releases the metal under oxidative conditions and acts as an Se scavenging oxidant. Zinc is involved in the regulation of glutathione metabolism and the overall protein thiol redox status [122]. Zinc competes with redox-active transition metals, iron and copper, for certain binding sites. When zinc binds to these sites, copper and iron are forced to undergo hydrolytic polymerization into unreactive structures, thereby prohibiting the catalysis of free radical formation and the initiation of lipid peroxidation [121,122,123]. Zinc is mainly expressed in the hippocampus, amygdala, cerebral cortex, thalamus, and olfactory cortex in the brain [124] and is stored as free zinc ions (Zn^2+^) in the presynaptic glutamatergic neurons. Zinc in synaptic vesicles is released with glutamate and acts as a potent extracellular modulator by interacting with many synaptic receptors during synaptic activity [123].

Co-treatment with zinc and Se significantly decreased mitochondrial dysfunction, ROS levels, and lipid peroxidation levels, while significantly increasing cognitive performance, SOD, glutathione peroxidase, and catalase activity in the mitochondria of the brain in an AD rat model [125]. In a double-blind, placebo-controlled trial of zinc supplementation for premenstrual syndrome, sixty women (18–30 years) were randomly assigned to receive either 30 mg of zinc gluconate and/or placebo for 12 weeks. The zinc-administered group showed beneficial effects on physical and psychological symptoms of premenstrual syndrome, total antioxidant capacity, and brain-derived neurotrophic factor [126].

Other essential metals, such as copper, iron, and magnesium, play an important role in the maintenance of cell homeostasis and preservation of life. They display important structural, regulatory, and catalytic functions in different types of proteins, such as enzymes, receptors, and transporters. Cu^+^ and magnesium are the cofactors for enzymes such as COX and/or supper zinc, SOD, and neuronal Cu enrichment predispose to Cu^2+^-catalyzed Fenton chemistry and H_2_O_2_-assisted protein oxidation. In particularly, iron continuously shifts between ferrous ion (Fe^2+^) and ferric ion (Fe^3+^) states in a redox reaction in the presence of O_2_. The constitution of iron in the body is in the form of 65% (Fe^2+^) ions bound to hemoglobin; less than 10% of ions are expressed with myoglobin (Fe^2+^), cytochromes (Fe^2+^ or Fe^3+^), and iron-containing enzymes, and 25% of the ions are bound to iron-storage proteins such as transferrin, ferritin, and hemosiderin [127]. Iron present in the cells in the reduced (Fe^2+^) and oxidized (Fe^3+^) states can serve both as an electron donor and electron acceptor. Particularly, the ferrous form of iron can act as a catalyst in the potentiation of oxygen toxicity by generating a wide range of free radical species, including hydroxyl radicals. In addition, an excessive amount of this essential metal induces toxicity, leading to pathological conditions generated by oxidative stress and neurodegeneration [128]. Although homeostasis of iron is essential for physiological functions in the brain, less than 2% of total body iron is present in the brain. Iron contributes to the activity of various enzymes involved in neurotransmitter synthesis and myelination of axons of motor neurons in the brain. Iron accumulation induces features of neurodegenerative disorders including AD, PD, HD, ALS, and neurodegeneration with brain iron accumulation (NBIA). The pathogenesis of neurodegenerative diseases shows a relationship with a dramatic increase in iron content in the brain, which is correlated with the production of ROS [127].

#### 3.2.2. ROS/RNS Scavengers (Vitamin C, E, and A)

##### Vitamin C

**Figure 3:**
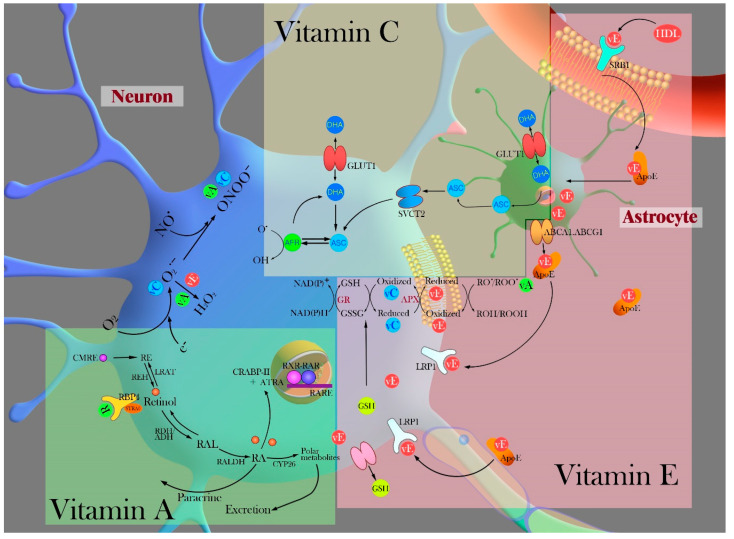
Schematic representation of vitamin uptake and protective mechanisms by exogenous vitamins as antioxidants. **Vitamin C:** During uptake in the CNS, ascorbate passes through the BBB to enter directly through the SVCT-2 and/or possibly DHA through GLUT1s. Moreover, the neuronal uptake of ascorbate occurs through SVCT-2 and DHA via the GLUT1s. In the neuron, DHA can be reduced to ascorbate or released back into the extracellular space by GLUT1. Ascorbate free radicals convert to form DHA and ascorbate. Ascorbate recycles both the ascorbate free radical and DHA by cellular metabolism. Astrocytes contain ascorbate from recycling of DHA which is taken up through GLUT1s. Neurons directly acquire ascorbate via SVCT-2. **Vitamin E:** With respect to vitamin E uptake, HDL particles can pass through SRB1 receptors expressed on endothelial cells. Astrocytes that exist adjacent to the BBB take up vitamin E into the inner cell membrane. Synthesized ApoE lipoproteins take up vitamin E that is left out of an ABC transporter, for transport into neurons through LRP1 requiring vitamin E for maintenance or during conditions of oxidative stress. **Vitamin A:** In cellular retinoid signaling pathways, retinol is metabolized to all-trans-retinoic acid (ATRA). Vitamin A (retinol, ROL) binds to plasma retinol binding protein (RBP4) and circulates; RBP4 protein binds to the membrane receptor STAR6 to promote cellular absorption of retinol from the cells. A chylomicron remnant (CMRE), as a form of circulating vitamin A, can serve as a source of vitamin A for the cells and retinol is esterified and stored by lecithin: retinol acyltransferase (LRAT) and is reversibly oxidized to retinaldehyde (RAL) by retinol dehydrogenase (RDH/ADH). In addition, retinol is further oxidized to RA in an irreversible manner by retinaldehyde dehydrogenase (RALDH). ATRA regulates gene transcription through retinoic acid receptors (RAR) and/or retinoid X receptors (RXRs) which are bound to retinoic acid response elements (RARE) in the nucleus. These representative schematics are modified from [130,154,161]. Abbreviations: VC, vitamin C; VE, vitamin E; VA, vitamin A; ASC, ascorbate; ASF, ascorbate free radical; DHA, dehydroascorbic acid; GLUT1, glucose transports; SVTC-2, sodium-dependent transporters; LRP, lipoprotein receptor-related protein; GSH, glutathione; ApoE, apolipoprotein E; HDL, density lipoprotein; CMRE, chylomicron remnant; CRABP, cellular retinoic acid-binding protein; LRAT, lethicin: retinol acyltransferase; RBP, retinol binding protein; RAL, retinaldehyde; RDH/ADH, retinol dehydrogenase; RALDH, retinaldehyde dehydrogenase; RXR, retinoid X receptors; RAR, retinoic acid receptors; ATRA, all-trans-retinoic acid; BBB, blood–brain barrier.

Ascorbate (ascorbic acid, AA), a ubiquitous water-soluble antioxidant and a cofactor for several enzymes, can inhibit the generation of ROS, directly scavenge ROS/RNS, and repair other oxidized scavengers [129]. ROS generation is limited by ascorbate through the inhibition of NOX and nNOS. It also helps in the regeneration of alpha-tocopherol from alpha-tocopheroxyl radical and repair of glutathione. The highest concentration of ascorbate is expressed in the brain and is involved in CNS homeostasis. Endogenous ascorbate exists in two biological forms, the deprotonated ascorbate anion and dehydroascorbate (DHA), which is the product of the two-electron reversible oxidation of ascorbate [129]. The mechanism of ascorbate uptake involves absorption of dietary ascorbate in the intestine by sodium-dependent transporter-1 (SVCT-1) and dissolution in the blood. Ascorbate enters the CNS by slow transport from the plasma to the cerebrospinal fluid across the choroid plexus epithelium. Ascorbate can easily enter the brain through the glucose transporter (GLUT1) when a considerable amount of DHA is present in the blood. Ascorbate or DHA in the cerebrospinal fluid enters the neuron via sodium-dependent transporter-2 (SVCT-2) or GLUT1 transporters [130,131]. Once they enter the neuron, DHA can be reduced to ascorbate or released by GLUT1. Ascorbate produces ascorbate free radicals as one electron donor, which is reduced back to ascorbate within the cells by NADH- and NADPH-dependent reductase. Glial cells obtain ascorbate by reduction of DHA through GLUT1, and ascorbate uptake does not involve SVCT-2, which is different from neurons [131]. DHA goes through GLUT1 slowly to enter the choroid plexus and astrocytes [132,133,134]. The rapid entry of ascorbate is driven by SVCT-2 through the blood-brain-barrier (BBB) to neurons, and ascorbate modulates SVCT-2 translocation to the plasma membrane, ensuring optimal ascorbate uptake in the neurons [135,136] (Figure 3). The oxidizing and free radical scavenging activity of ascorbate inside the cell is not limited to the aqueous phase, but also includes protection of membranes and other hydrophobic compartments through interaction with vitamin E [137]. Aging decreases the extracellular concentration and the uptake of ascorbate in the brain, which is consistent with an increase in oxidative stress. The neurobiological role of ascorbate in the brain is seen in neuromodulation and neuroprotection [129]. In the role of a neuromodulator, ascorbate release is involved in the uptake or clearance of glutamate from the synapse after its release from the axon terminals. Specifically, L-glutamate promotes ascorbate release as a consequence of removing glutamate from the synapse [138]. Although ascorbate does not act as a classical neurotransmitter, extracellular ascorbate may influence neurotransmission. In particular, ascorbate directly modulates neural excitability through inhibition of T-type Ca^2+^ channels [139], participates in the reduction of extracellular oxidants, which effects the redox status of catecholamines [140], and influences the release of biogenic amines in striatum [141] and pituitary neuropeptides [142]. Another neuroprotective role of ascorbate is the attenuation of neurotoxicity, which results from the scavenging activity [143]. Ascorbate inhibits the oxidative stress triggered by various neurotoxins and protects against ethanol-induced apoptotic neurodegeneration in prenatal rat hippocampal neurons [144]. Oxidative stress in stroke, hypoxia, ischemia, and seizure activity leads to massive glutamate release and subsequent excitotoxicity, a result of over-activation of glutamate receptors [145]. Therefore, ascorbate can protect against glutamate-induced excitotoxicity and neurodegeneration.

Many researchers have reported that neurodegeneration can be reversed or lessened by ascorbate treatment [146,147,148]. From the viewpoint of the effect of aging, the extracellular concentration and uptake of ascorbate is decreased in the brain, which is consistent with an increase in oxidative stress [149]. Moreover, excess ascorbate intake or deficiency may influence brain aging [150]. Thus, careful maintenance of ascorbate levels in the brain may be important during the life span. There have been extensive reviews on the role of ascorbate in the brain with a focus on neurodegenerative disease [130,131]. Even though ascorbate has a complicated interaction with the neurotransmitter system, ascorbate is considered relevant for use as an antioxidant therapy because neurons are sensitive to ascorbate deficiency and excess oxidant stress. In our previous work [151], we studied the protective effects of AA and DHA on KA-induced oxidative stress using organotypic hippocampal slice cultures. After 12 h of KA treatment, significant delayed neuronal death was detected in the CA3, but not in the CA1 region. Pretreatment with intermediate doses of AA and DHA significantly prevented cell death and reduced ROS levels, as well as mitochondrial dysfunction in the CA3 region. However, pretreatment with high doses of AA or DHA was not effective. Attempting to elevate the brain ascorbate by the systemic administration of high doses of ascorbate is very difficult. The level of AA in the extracellular fluid of the striatum was decreased in a transgenic mouse model of HD; hence, restoring striatal extracellular AA levels with high doses of ascorbate improved behavior [152,153].

##### Vitamin E

Vitamin E is a major group of lipid-soluble antioxidants called tocopherols and tocotrienols, of which the most biologically active isoform is α-tocopherol [154]. It is a major chain-breaking antioxidant and exists in a low molar ratio compared to unsaturated phospholipids. The most important function of vitamin E is its antioxidant activity, which protects the integrity of cellular membranes from polyunsaturated fatty acid-generated oxygen free radicals and to act as a direct scavenger of superoxide and hydroxyl radicals [154,155]. Based on studies of brain capillary endothelial cells, the mechanism of entry of α-tocopherol into the CNS correlated to α-tocopherol and scavenger receptor class B type 1 (SRB1) levels. α-Tocopherol uptake occurs via a selective high-density lipoprotein (HDL) pathway, which modulates the expression of SRB1 receptor [156,157,158]. Once α-tocopherol passes through the BBB, it may be directly delivered to specialized astrocytes. Astrocyte-synthesized apolipoprotein E (ApoE) moves through the cerebral spinal fluid transporting α-tocopherol between various cell types in the CNS cell [154,159]. ApoE lipoprotein particles in astrocytes are secreted through membrane proteins and interact with low-density lipoprotein receptor-related protein (LRP) on the neurons [160]. The neurons take up these ApoE particles and distribute them throughout the body, axon, and dendrites to preserve the membrane from lipid peroxidation (Figure 3). The expression of α-tocopherol transfer protein is enhanced by an increase in ROS, and it is useful for combating neuronal damage.

The antioxidant ability of vitamin E is continuously restored via vitamin E recycling by other antioxidants such as vitamin C, ubiquinols, and thiols. The half-life of vitamin E in the brain tissue is slower than that of other vitamins, and it is also actively retained and protected from auto-oxidation in the brain [162]. The distribution of α-tocopherol is significantly different in the brain. These CNS-regional disparities are suggestive of the specific protective antioxidant effect of α-tocopherol. Specifically, the concentrations of α-tocopherol were relatively higher in the nuclear membranes than in the other membranes of the brain. It has been suggested that α-tocopherol may play a role in nuclear-associated functions in the cerebellum and striatum, wherein preferential accumulation of α-tocopherol in the membrane was most apparent [163]. Vitamin E, similar to other radical scavengers/trappers, influences the flux of lipid hydroperoxide (LOOH), which is derived from both spontaneous and enzymatic formation of lipid peroxyl radicals (LOO^•^) on the cellular membrane [164]. The effects of vitamin E on peroxidation activity appear to involve both the radical scavenging mechanism such as the H atom donor activity and a physical interaction with the polyunsaturated lipid substrate. Tocotrienols, another form of vitamin E that are highly metabolized, show more potent inhibition of the phospholipase A2/lipoxygenases pathway as compared to tocopherols, and have different cellular bioavailability, distribution, and protein interaction in the saturated and unsaturated form of vitamin E in the brain [165]. Deficiency of vitamin E showed increased biochemical and histological markers of oxidative stress, including total glutathione and lipid peroxidation in the CNS.

**Figure 4:**
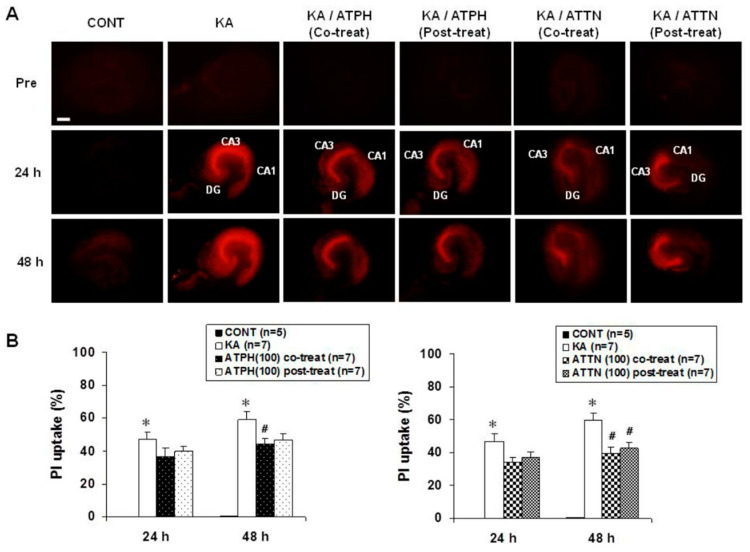
Neuroprotective effect of vitamin E. (**A**): Representative propidium iodide (PI) images. When hippocampal slices were exposed to 5 μM KA for 15 h, PI uptake in the CA3 region was significantly higher than the CA1 region. Co-treatment using ATPH (100 μM) or ATTN (100 μM) with KA significantly reduced PI uptake in the CA3 region compared with KA treatment alone. (**B**): Quantification of PI intensity. * *p* < 0.05, # *p* < 0.05; one-way ANOVA followed by Dunnett’s post hoc comparison (* *p* < 0.05 vs. normal, # *p* < 0.05 vs. KA-treated cultures). This present data is a part of our previous research showing neuronal rescue after oxidative stress by alpha-tocopherol and tocotrienol treatment [166]. Abbreviations: ATPH, alpha-tocopherol; ATTN, alpha-tocotrienol; OHSC, organotypic hippocampal slice culture; KA, kainic acid; PI, propidium iodide; DCF, dichlorofluorescein.

Therapeutic effects of α-tocopherol by application of vitamin E in neurological lesions caused by neuronal excitotoxicity and the conditional activation of neuroglial cells have also been reported. In KA treatment-induced oxidative stress [166], delayed neuronal death was detected in the hippocampal CA3 region and ROS formation and lipid peroxidation were also increased. Both co-treatment and post-treatment with α-tocopherol (100 µM) or α-tocotrienol (100 µM) significantly increased cell survival and reduced the number of TUNEL-positive cells in the CA3 region. Increased dichlorofluorescein (DCF) fluorescence and thiobarbiturate reactive substance (TBARS) levels were decreased by drug treatment (Figure 4). In AD patients, long-term administration of vitamin D and E alone or in combination could inhibit morphological changes of neurons and improve learning and memory [167]. Vitamin E prevented the memory impairment associated with post-traumatic stress disorder (PTSD)-like behavior in rats. Significant decreases in oxidative stress biomarkers were detected with reduced glutathione/oxidized glutathione (GSH/GSSG) ratio [168]. In PD patients, omega-3 fatty acid and vitamin E co-supplementation had favorable effects on the UPDRS score and increased the total antioxidant capacity (TAC) and GSH concentration compared to placebo [169].

##### Vitamin A

Vitamin A, carotenoids including retinol and beta-carotene, are fat-soluble chemicals synthesized by plants and some microorganisms and have many functions in human growth, development, and health [170,171]. Vitamin A is available in the human diet as pro-vitamin A carotenoids and preformed vitamin A (retinol-alcohol form, retinal-aldehyde form, retinoic acid-carboxylic acid form, and retinyl ester-ester form). The preformed vitamin A from animal-derived food and pro-vitamin A carotenoids from plant-derived foods are converted to all-trans-retinol as vitamin A alcohol by a series of reactions in the intestine. Carotenoids, mainly via dietary intake, can function directly as antioxidants by quenching ROS through energy transfer [172]. Vitamin A deprivation was investigated in the deficiency of cognitive function in adult mice and rats, which highlights the importance of adequate vitamin A status by the retinoid signaling pathways [161]. Carotenoids are classified into pro-vitamin A carotenoids such as β-carotene and β-cryptoxanthin, which are capable of converting to retinal, and non-pro-vitamin A carotenoids such as lycopene and lutein, which cannot be converted to retinal [173]. Carotenoids act through several pathways and interact with free radicals in the plasma, mitochondria, and nuclear membranes of cells via electron transfer, hydrogen abstraction, and physical quenching [174]. Carotenoids indirectly react with cell signaling cascades, including the nuclear factor elytroid 2 (NF-E2)-related factor 2 (Nrf2), NF-κB, or mitogen-activated protein kinase (MAPK) [175,176]. The antioxidant action of carotenoids involves singlet oxygen quenching and trapping of peroxyl radicals. Retinol-binding proteins observed in the BBB regulate the access of retinol into the brain [177]. High concentrations of retinol and carotenoids have been observed in the postmortem human frontal lobe cortex [178]. β-carotene, a precursor of retinol and retinoic acid, is reported to be a potent free radical quencher, singlet oxygen scavenger, and lipid antioxidant in tissues and plasma. Therefore, β-carotene acts in a hydrophobic environment such as the lipid core of the membranes and is used faster than α-tocopherol, implying that β-carotene is more favorable than α-tocopherol to quench lipophilic radicals in the membrane. The most efficient synergistic inhibition during oxidative stress was observed with a combination treatment of α-tocopherol and ascorbic acid [179].

Retinoids, compounds structurally related to vitamin A, are considered vitamin A derivatives that contribute to regular cellular morphogenesis, proliferation, and differentiation. Retinoids are involved in normal signaling cascades in modulating brain functions [180]. Retinoids modulate the availability of glucocorticosteroids in the brain, an important biological mechanism that can be explored in many stress-related pathologies to prevent alterations in the plasticity of the hippocampus [181]. Retinol metabolic pathways have shown that retinol can be stored intracellularly as retinyl esters and metabolized into all-trans-retinoic acid (ATRA) as a bioactive derivative. ATRA induces cellular differentiation and growth by reacting to retinoic acid receptors (RARs). Cellular retinol-binding proteins (CRBP-I and II) and cellular retinoic acid-binding proteins (CRABP-I, II) are distributed in the adult CNS. Furthermore, CRBP-I distribution parallels that of ATRA with expression in the meninges, hippocampus, amygdala, and olfactory bulb [182] (Figure 3). Under oxidative stress conditions such as metal exposure and production and accumulation of ROS, retinoids protect the cells against this imbalance through multiple mechanisms, including interference with ROS production, scavenging free radicals directly, upregulation of antioxidant enzymes, and signaling pathways involved in defense system such as Nrf2 signaling [183]. It has also been observed that retinoic acid has a protective effect on neuronal apoptosis and oxidative damage by reducing glutathione [184] and restoring SOD-1 and SOD-2 in the hippocampal cells [185]. The role of retinoid signal transduction in the control of dopaminergic neurotransmission was observed in the presence of high levels of retinoic acid-synthesizing enzymes [186] and RAR, which may play a critical role in controlling the survival, adaptation, and homeostatic regulation of the dopaminergic system [187]. Retinoid signaling play a physiological role in synaptic plasticity and learning and memory behaviors [188].

Retinoic acid supplementation upregulated μ-type opioid receptor 1 (MOR1) and its signaling and alleviated dyskinetic movements, which is a known consequence of prolonged administration of L-DOPA, in Pitx3^ak/ak^ mice [189]. Moreover, RA triggered the neuroprotective effect on DA neurons in MPTP-treated mice model of PD. Administration of RA-loaded polymeric nanoparticle significantly reduced the loss of DA neuron in the substantia nigra as well as their neuronal fiber/axonal innervations in the striatum [190]. Moreover, oral administration of lycopene (5–20 mg/kg), a carotenoid with unique pharmacological properties, attenuated oxidative stress in mice with PD, which was induced with intraperitoneal injection of 1-methyl-4-phenyl-1,2,3,6-tetrahydropyridine (MPTP). Lycopene supplementation also inhibits apoptosis in PD mice by decreasing Bax and caspases, and contrarily increasing Bcl-2 [191]. Dietary supplementation with astaxanthin, another carotenoid family member, significantly decreased intracellular ROS accumulation in a hippocampal neuronal cell line after exposure to glutamate and induced antioxidant mediators, such as heme oxygenase-1 (HO-1) and nuclear Nrf2 expression in vitro [192]. In multiple sclerosis (MS) patients, vitamin A supplementation had a significant effect in the treatment group for fatigue and depression. In addition, when a synthetic retinoid was tested, it showed reduction in inflammation, Aβ burden, and tau phosphorylation with associated cognitive benefits in AD patients [193,194].

Aside from elucidating the efficiency of endogenous antioxidants, several studies have increasingly accredited the role of various exogenous antioxidants. Limited clinical studies are reported herein and are illustrated in Table 1. Table 1 also shows several examples of previously mentioned exogenous antioxidant reactions that have worked in clinical settings. Although the administration of exogenous antioxidants showed positive effects, there are some inconsistencies in clinical trials. The clinical trials performed multivariable analyses with various factors, such as sample size, replication or validation studies using the same agent and outcome measures, assessment of vitamins, different conditions of endogenous antioxidants, long-term monitoring, etc., which caused variability because of inconsistencies. In general, several common antioxidants and their clinical effects are described in Table 1.

#### 3.2.3. Nrf2 Antioxidant System

The transcription factor Nrf2 is characterized as a regulator of redox homeostasis and antioxidant defense mechanisms. This protective pathway also encompasses the activation of a detoxification network such as oxidation/reduction factors (Phase I), metabolizing enzymes (Phase II), efflux transporters (Phase III), and free radical scavengers [201]. Oxidation/reduction factors consisting of nearly 500 genes encoding proteins, including redox balancing factors, stress response proteins, detoxifying enzymes, and metabolic enzymes such as NAD(P)H quinone oxidoreductase (NQO1), HO-1, SOD, GST, GSR, GSH-Px, carbonyl reductase (CR), and glutamate-cysteine ligase (GCL) play important roles in antioxidant and pro-survival effects and detoxification of xenobiotics. Nrf2 is generally targeted for ubiquitin-mediated degradation by its endogenous inhibitor Keap1, but oxidative modification of Keap1 inhibits the Nrf2 degradation process during conditions of redox imbalance [202]. To maintain cellular redox homeostasis, basal Nrf2 accumulation increases to mediate the normal expression of antioxidant response element (ARE)-dependent genes in the nucleus. The mitochondrial membrane directly interacts with Nrf2 which can respond to mitochondrial oxidative stressors that can collapse cellular bioenergetics leading to cell apoptosis [203]. Thus, the Keap1/Nrf2 system is another good homeostatic regulator of intrinsic cellular antioxidant defense and mitochondrial health. The suppression of Nrf2 activity increases the susceptibility of the brain to the damaging effects of oxidative stress and inflammatory stimuli [204]. Nrf2 activation promotes neuroprotective effects in both in vitro and in vivo neurodegenerative models. Nrf2 activity diminishes with age, and consequently, the effect of antioxidant enzyme activity decreases. Nrf2/ARE system impairment leads to higher susceptibility to oxidative injury, abnormal protein aggregation and neurodegeneration in the brain. Many studies have demonstrated the importance of the Nrf2/ARE pathway in the pathogenesis and control of neurological disorders, including PD, AD, ischemia, and other neurodegenerative diseases.

After middle cerebral artery occlusion (MCAO) in rats, Keap1 levels are decreased and this loss is correlated with an increase in Nrf2 and its downstream proteins, such as thioredoxins, GSH synthases, and HO-1 [205]. Stroke models of Nrf2-deficient mice exhibit higher levels of ROS than the wild-type littermates, which supports the natural compensatory mechanism of Nrf2 [206]. In AD transgenic mice, Nrf2-deficiency brains presented increased marker of oxidative stress and exhibited deficits in spatial learning and memory [204]. Nrf2 knockout and wild-type mice can be administered MPTP with doses ranging from 20 to 60 mg/kg for an animal model of PD. Nrf2^−/−^ mice exhibited increased sensitivity to the dopaminergic toxins MPTP and 6-OHDA [207]. Nrf2 deficiency increased the MPTP sensitivity by 30 mg/kg administration of MPTP, but astrocytic Nrf2 overexpression showed the amelioration of MPTP toxicity in ARE-hPAP mice [208]. Astrocytic Nrf2 reduced the MPTP neurotoxicity by β-lapachone treatment in PD models [209].

## 4. Conclusions

Brains are metabolically active and have a high demand for large amounts of ATP to maintain their physiological function. Additionally, the brain has a relatively low level of antioxidants, low repair capacity, non-replicating nature of neuronal cells, and a high ratio of membrane surface to cytoplasm. ROS production is largely through oxidative phosphorylation and increased free radicals play a central role in neurological disorders by the imbalance of pro-oxidant and antioxidant agents in the brain. This underpins the importance of targeting antioxidant systems to counteract the oxidative stress and associated brain diseases. Indeed, the antioxidant system is important for the rescue of neuronal cells from oxidative stress and preservation of the right redox balance in the brain tissue by promoting antioxidative defenses for neutralizing ROS and by blocking transcription. Currently, there is growing research interest in the development of new/combination exogenous supplementation of antioxidants, retention of the functional integrity of intrinsic antioxidant systems for preventing harmful CNS disorders, and identification of novel approaches to therapy to prevent and/or reduce brain injury. Thus, high levels and variable metabolites in the brain indicate an increased requirement for antioxidant defense systems as enzymatic and non-enzymatic molecules. Redox biology fulfills important physiological functions that extend beyond its role on oxidative stress, such as cell signaling [210,211]. Perhaps, ROS generation may be necessary for cell function. Although their role is still ill-defined, the antioxidant systems may contribute to the controlled release of ROS for normal brain function.

## Figures and Tables

**Table 1:** Clinical trials of antioxidant in neurodegenerative diseases.

Antioxidant	Number of Patients	Follow up Period	Dosage	Route	Effects	Disease	Reference
CoQ10	609	60 month	2400 mg/day	Oral	No	Huntington	[119,195]
	40	96 week	300 mg/day	Oral	Y	Parkinson	
Selenium	7540	7 year	200 µg/day	Oral	No	Alzheimer	[107,196]
		6 month	1000 µg/day	I.V.	Y	Traumatic brain injury	
Zinc	43	12 week	20 mg/day	Oral	Y	Depression with multiple sclerosis	[197]
Vitamin A	50	28 day	30 mg/day	Oral	Y	Alzheimer	[191,198]
	101	6 month	25,000 UI/day	Oral	Y	Multiple sclerosis	
Vitamin C	12	6 day	2 g/day	Oral	Y	Hyperoxia	[147,199]
	60	3 month	500 mg/day	Oral	Y	Trauma surgery	
Vitamin E	7540	7 year	400 UI/day	Oral	No	Alzheimer	[168,196,200]
	60	3 month	400 UI/day	Oral	Y	Parkinson	
	50	12 month	45 UI/day	Oral	Y	Parkinson	

## Abbreviations

AD	Alzheimer disease
ALS	amyotrophic lateral sclerosis
ApoE	apolipoprotein E
ARE	antioxidant response element
ATP	adenosine triphosphate
ATPH	alpha-tocopherol
ATRA	all-trans-retinoic acid
ATTN	alpha-tocotrienol
Aβ	beta-amyloid
CA3	cornu ammonis 3
CAT	catalase
CNS	central nervous system
CoQ	coenzyme Q
COX	cyclooxygenase
CR	carbonyl reductase
CRABP	cellular retinoic acid-binding proteins
CRBP	cellular retinol binding proteins
DCF	dichlorofluorescein
DHA	dehydroascorbate
EC-SOD	extracellular superoxide dismutase
FADH2	dihydroflavine-adenine dinucleotide
Fe^2+^	ferrous ion
Fe^3+^	ferric ion
GCL	glutamate-cysteine ligase
GLUT1	glucose transports
GPx	glutathione peroxidase
GR	glutathione reductase
GSH	glutathione
GSSG	glutathione disulfide
hAPP	human amyloid precursor protein
HD	Huntington’s disease
HDL	high density lipoprotein
HO-1	heme oxygenase-1
iNOS	inducible nitric oxide synthase
KA	kainic acid
LRP	lipoprotein receptor-related protein
MAPK	mitogen-activated protein kinase
MCAO	middle cerebral artery occlusion
Mn-SOD	manganese containing superoxide dismutase
MOR	μ-type opioid receptor
MPTP	1-methyl-4-phenyl-1,2,3,6-tetrahydropyridine
NADH	reduced form of nicotinamide adenine dinucleotide
NADPH	reduced form of nicotinamide adenine dinucleotide phosphate
NBIA	neurodegeneration brain iron accumulation
NF-κB	nuclear factor-κB
nNOS	neuronal nitric oxide synthase
NOX	NADPH oxidase
NQO1	NADPH quinone oxidoreductase
Nrf2	nuclear factor elytroid 2-related factor 2
6-OHDA	6-hydroxydopamine
OHSC	organotypic hippocampal slice culture
PD	Parkinson disease
PI	propidium iodide
PTSD	post-traumatic stress disorder
RAR	retinoic acid receptors
RNS	reactive nitrogen species
ROS	reactive oxygen species
Se	selenium
SOD	superoxide dismutase
SRB1	scavenger receptor class B type 1
SVCT	sodium-vitamin C co-transporters
TAC	total antioxidant capacity
TBARS	thiobarbiturate reactive substance
Trx	thioredoxin
TrxR	thioredoxin reductase
UV	ultra violet
